# DamID as a versatile tool for understanding gene regulation

**DOI:** 10.1242/dev.173666

**Published:** 2019-03-15

**Authors:** Gabriel N. Aughey, Seth W. Cheetham, Tony D. Southall

**Affiliations:** 1 Department of Life Sciences, Imperial College London, Sir Ernst Chain Building, London, SW7 2AZ, UK; 2 Mater Research Institute-University of Queensland, TRI Building, Woolloongabba, QLD 4102, Australia

**Keywords:** DamID, Chromatin, Gene regulation, Genomics

## Abstract

The interaction of proteins and RNA with chromatin underlies the regulation of gene expression. The ability to profile easily these interactions is fundamental for understanding chromatin biology *in vivo*. DNA adenine methyltransferase identification (DamID) profiles genome-wide protein-DNA interactions without antibodies, fixation or protein pull-downs. Recently, DamID has been adapted for applications beyond simple assaying of protein-DNA interactions, such as for studying RNA-chromatin interactions, chromatin accessibility and long-range chromosome interactions. Here, we provide an overview of DamID and introduce improvements to the technology, discuss their applications and compare alternative methodologies.

## Abstract (precis)

**Summary:** This Primer provides an overview of DNA adenine methyltransferase identification (DamID), which is used to profile genome-wide chromatin interactions, and introduces recent improvements to the technology.

## Introduction

Techniques that identify the binding of regulatory factors to chromatin are essential for uncovering the mechanisms that maintain and control gene expression. Chromatin immunoprecipitation (ChIP) is the most widely used method to identify the chromatin-binding sites of proteins and has been instrumental in advancing our understanding of gene regulation (Visa and Jordán-Pla, 2018). In 2000, Bas van Steensel and Steven Henikoff pioneered the use of DNA adenine methylase identification (DamID) as an alternative to ChIP (van Steensel and Henikoff, 2000). DamID uses *E. coli* adenine methyltransferase (Dam) fused to a protein of interest, which is expressed at low levels. When the fusion protein comes into close proximity with DNA, Dam methylates adenines (m6A) in nearby GATC motifs. These methylated sequences can be enriched using a methylation-sensitive endonuclease (DpnI) and ligation-mediated PCR. The resulting DNA is then sequenced to produce genome-wide binding profiles for the chromatin-interacting protein of interest (see Box 1). Usually, a control sample is also produced in which untethered Dam methylase has been expressed. When the data is analysed, the Dam-fusion is normalised against the background methylation from the Dam-only control.

Box 1. DamID experimental pipeline The basic DamID experimental pipeline is outlined below, and is largely similar in many of the newly developed variants.

- Generation of animals or cells with Dam-fusion transgene and Dam-only control.
- Extraction of genomic DNA from tissue or organism of interest.
- Digestion of DNA with DpnI (digests methylated GATC motifs only).
- Ligation of PCR adapters to digested DNA.
- Digestion of remaining unmethylated DNA with DpnII (digests unmethylated GATC motifs only).
- PCR amplification of adapter ligated GATC fragments.
- Sequencing library preparation using standard protocols.

Detailed protocols have been published for DamID (Vogel et al., 2007) and targeted DamID (Marshall et al., 2016; Tosti et al., 2018). Variations on this basic protocol have also been described (Gutierrez-Triana et al., 2016) and bioinformatic tools for the analysis of DamID data are readily available (Li et al., 2015; Marshall and Brand, 2015).

Since its inception, DamID has been used to detect protein-DNA interactions in a range of experimental organisms including *Drosophila* (van Steensel and Henikoff, 2000), *C. elegans* (Schuster et al., 2010), *Arabidopsis* (Germann et al., 2006), medaka (Gutierrez-Triana et al., 2016), mice (Tosti et al., 2018) and human cells (Vogel et al., 2006). DamID is an increasingly versatile tool and has recently been adapted in inventive ways to elucidate the molecular mechanisms of gene regulation. Here, we review the recent advancements in DamID technologies, which move beyond protein-DNA interactions towards the interrogation of an increasingly broad range of chromatin features *in vivo.*

## DamID versus ChIP

Although ChIP has been the predominant chromatin profiling method for many years, DamID offers a range of advantages for some applications. Previous reviews have compared the relative merits of DamID with ChIP (Aughey and Southall, 2016; Otsuki et al., 2014); however, in this section we will provide an overview of these considerations.

ChIP relies on immunoprecipitation using an antibody to target the protein of interest and sequencing of the co-precipitated DNA fragments. The primary advantage of DamID is that there is no requirement for antibodies or other affinity reagents to purify the protein of interest. Instead, transgenic cells must be produced in which Dam is expressed. DamID is, therefore, limited to genetically tractable model systems. One of the most important differences between the two techniques is that ChIP, and its derivatives, provide a snapshot of protein occupancy at a single point in time. DamID, however, typically relies on DNA methylation that occurs over a period of several hours. Therefore, ChIP may be better suited to a particular research question depending on the temporal resolution required. However, DamID has the advantage of giving an indication of chromatin-binding events that occur *in vivo*, in contrast to ChIP, which assays the interaction after cell and/or tissue processing. Similarly, the spatial resolution differs between the two, with ChIP having much greater binding resolution, whereas DamID is limited by GATC motif availability.

Although DamID represents an alternative to chromatin purification-based methods, it also provides a powerful complementary approach to independently verify important results. Although ChIP-seq has been the gold standard for the identification of genome-wide transcription factor-binding sites, there is growing evidence that some identified targets may be the result of experimental artefacts. Chromatin-associated proteins are often erroneously found to associate with unrelated highly expressed gene promoters in ChIP-seq experiments (Jain et al., 2015; Park et al., 2013; Teytelman et al., 2013). These so-called ‘phantom peaks’ are difficult to distinguish from bona fide binding sites. They may appear in ChIP experiments that have been performed with different antibodies and they remain upon ablation of the target gene (Jain et al., 2015). Before DamID was developed, the genome-wide profiles of transcription factor binding that had been determined by ChIP could not be directly confirmed using an alternative approach. Multiple studies have directly compared DamID with ChIP binding profiles, all of which show strong similarity between the two methods (Cheetham et al., 2018; Moorman et al., 2006; Nègre et al., 2006; Southall et al., 2013).

Modifications to both ChIP and DamID have been developed that address some of their respective limitations or adapt their use for more specialised applications (see Visa and Jordán-Pla, 2018 for more information on ChIP). For example, CUT&RUN (in which micrococcal nuclease is used to cleave DNA specifically at antibody-bound target sites) is becoming an increasingly popular alternative to ChIP (Skene and Henikoff, 2017). This method also does not require chemical crosslinking and has much greater signal-to-noise than basic ChIP. Given the key differences that are inherent to ChIP- or DamID-related techniques, the choice of the best method for a given research objective must be carefully considered.

## Cell-type-specific DamID approaches

Cell-type-specific regulation of gene expression underlies the growth and development of complex animal tissues. Whereas assaying DNA-protein interactions in cell lines or homogeneous tissues is relatively straightforward, determining protein-binding profiles in a specific cell type within a complex tissue is considerably more challenging. Physical isolation of the cells of interest can be laborious, inefficient and usually requires large amounts of tissue (Adan et al., 2017). Furthermore, tissue dissociation protocols can result in gene expression artefacts (van den Brink et al., 2017). To address these problems, two DamID methods that allow cell-specific profiling of DNA binding without cell isolation have been developed.

**Fig. 1.**
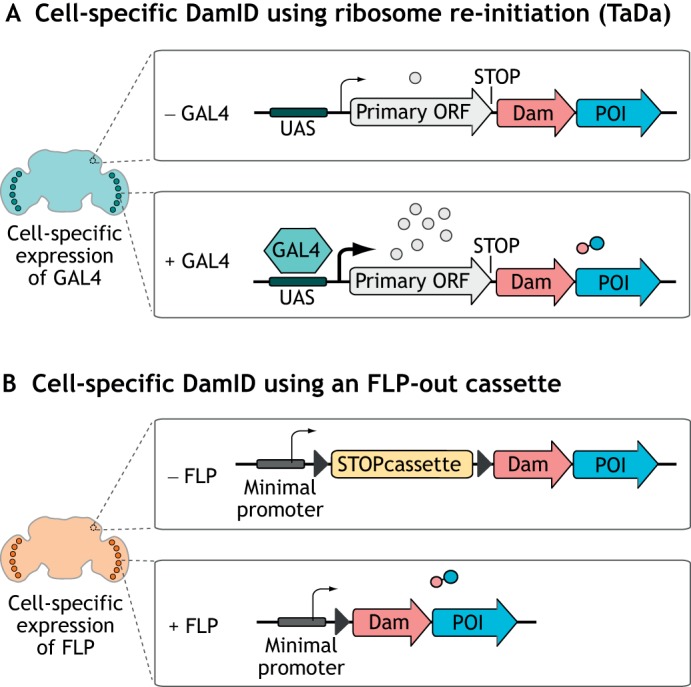
**Methods for cell-specific DamID profiling.** (A) TaDa allows cell-specific low-level expression of Dam by attenuating its translation using an upstream ORF. (B) Cell type-specific expression of FLP can remove a stop cassette from in between a minimal promoter and the Dam ORF, allowing low-level expression of Dam in the cells of interest.

Dam expression must be kept at exceptionally low levels to avoid saturating methylation and toxicity (see Box 2). Conventional DamID exploits a ‘leaky’ basal promoter to allow low-level expression of Dam (van Steensel and Henikoff, 2000). Cell-specific Dam expression can be achieved in several ways. One approach, Targeted DamID (TaDa), attenuates Dam levels using the expression of a bicistronic construct in which the Dam sequence is placed downstream of a primary open reading frame (ORF) (Southall et al., 2013) (Fig. 1A). The expression of Dam at appropriate levels relies on translational re-initiation of the ribosome between the ORFs (Kozak, 2001; Luukkonen et al., 1995). When this construct is expressed, the primary ORF is expressed at relatively high levels, whereas ribosome re-initiation occurs very infrequently at the secondary ORF (Dam-fusion), which results in very low levels of Dam translation. Crucially, expression can be induced solely in cells of interest using the *Drosophila* Gal4/UAS system, which results in targeted methylation in only the desired tissues. As the primary ORF usually encodes a fluorescent protein, this can be used for confirmation of expression in the desired cell type.

Box 2. Dam-related toxicity Expression of Dam at low levels is required to avoid saturating levels of methylation. However, limiting the levels of Dam protein may also protect against toxicity in some cell types. Although Dam may be expressed in at least some mammalian cell types in culture without any apparent side effects (Bas Van Steensel, personal communication), phenotypes have been reported following expression in the *Drosophila* developing nervous system (Southall et al., 2013) and in medaka embryos (Gutierrez-Triana et al., 2016). DNA adenine methylation (m6A) is widespread in bacteria, but for a long time it was assumed to be absent in eukaryotes. Several recent studies have overturned this view, describing m6A as an epigenetic mark in multicellular eukaryotes including *C. elegans* (Greer et al., 2015), *Drosophila* (Zhang et al., 2015) and mouse (Wu et al., 2016). Although these marks are typically only present at very low levels (<0.1% of bases), they may have an impact on gene expression. The mechanism of toxicity from Dam overexpression is unknown; however, it is feasible that the presence of high levels of m6A has deleterious effects on cell function when recognised by endogenous gene regulatory machinery. Alternatively, adenine methylation may interfere with DNA replication, transcription or any other fundamental process in ways that are difficult to predict. Toxic effects of transgenic Dam have not been studied extensively, therefore it is not possible to say which cells are likely to be affected owing to the lack of published data. Further study of this issue will be required to fully understand the effects of ectopic Dam expression.

An alternative approach to control Dam expression in *Drosophila* takes advantage of the FLP recombinase and its cognate ‘FRT’ recombination sites, which are frequently used to generate genetic mosaics (Griffin et al., 2014). This method incorporates a transcriptional terminator sequence that is flanked by FRT recombination sites, which is removed by FLP-mediated recombination in the cells of interest (Pindyurin et al., 2016) (Fig. 1B). Dam is then continuously expressed under the control of a ‘leaky’ heat-shock promoter.

The choice of technique to employ will depend on the experiment in question. If precise temporal control is desired in Gal4-driven experiments, Gal80 may need to be present to repress Gal4 activity. Another consideration is that the FLP-inducible technique causes an irreversible genetic change, ensuring that all progeny cells in a lineage will express the functioning Dam-fusion. Therefore, this approach is not suitable to determine the binding sites of proteins in precursor cell types during development. However, both methods result in methylation in specific cells of interest that can then be detected using methylation-sensitive PCR, without the requirement for cell sorting.

In addition to its use in *Drosophila*, TaDa has also been used to study tissue-specific binding of transcription factors in early mammalian embryonic development (Cheetham et al., 2018; Tosti et al., 2018). Mammalian Targeted DamID (MaTaDa) combines TaDa with the Cre-lox system to enable the identification of dynamic and cell-type-specific chromatin interactions (Cheetham et al., 2018). Importantly, MaTaDa is ultra-sensitive and can accurately map genome-wide transcription factor occupancy in as few as 1000-10,000 cells (Cheetham et al., 2018; Tosti et al., 2018).

As well as assaying transcription factor binding, cell-type-specific DamID techniques can be used for transcriptome profiling. By fusing Dam with components of the core RNA polymerase II (Pol II) transcription complex, methylation of all transcribed loci can be detected (Southall et al., 2013). As Pol II occupancy is reflective of active transcription, TaDa can be used as an alternative to isolation of cells and/or nuclei followed by RNA-seq to determine differences in gene expression in a cell type of interest (Marshall and Brand, 2017; Widmer et al., 2018), despite it not always being an exact reflection of the cytoplasmic mRNA levels (owing to post-transcriptional regulation). Together, the addition of these methods to the developmental biologist's toolbox greatly expands the potential for interrogation of chromatin interactions *in vivo*.

## Determining chromatin states with DamID

**Fig. 2.**
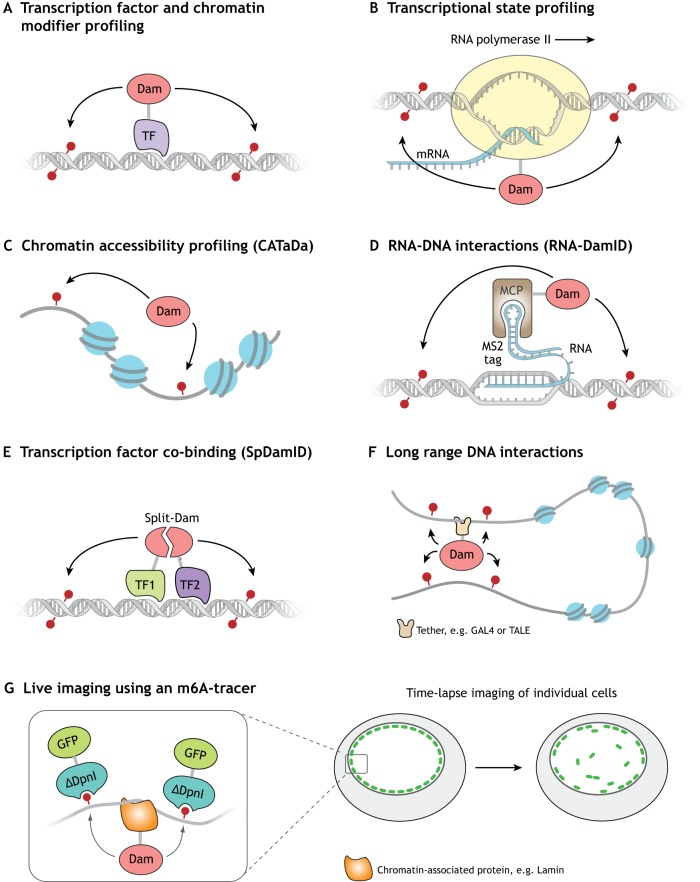
**DamID as a versatile tool for studying genome biology.** (A) Profiling of transcription factors (TFs) and chromatin-modifying enzymes. (B) Analysis of transcription by profiling Pol II occupancy. (C) Profiling of chromatin accessibility using untethered Dam (CATaDa). (D) Profiling the interaction of non-coding RNAs with DNA using an MCP-Dam fusion (RNA-DamID). (E) Identification of transcription factor co-binding using split DamID. (F) Identification of specific long-range DNA interactions by tethering Dam to a specific locus. (G) Live imaging of DNA that has associated with a specific protein (e.g. lamin).

Identification of genomic regions that are accessible to extrinsic factors is useful for uncovering regulatory DNA sequences. Recently, chromatin accessibility TaDa (CATaDa), a DamID-based approach to find regions of open chromatin, has been developed (Aughey et al., 2018). Whereas tethering Dam to a transcription factor (Fig. 2A) or Pol II (Fig. 2B) specifically methylates protein-binding sites, CATaDa relies on the expression of an untethered Dam, which results in methylation of all accessible regions (Fig. 2C). CATaDa detects similar genome-wide chromatin-accessibility profiles to ATAC-seq or FAIRE-seq in *Drosophila* eye discs. The major advantage of CATaDa is that Dam can be expressed cell-type-specifically, which enables the determination of chromatin accessibility for any cell type in a heterogeneous tissue without cell-isolation. CATaDa profiling of progenitor and differentiated cell types in the developing *Drosophila* CNS has identified enriched motifs that correspond to neural transcription factor-binding sites, and cell-type-specific enhancers (Aughey et al., 2018). Therefore, CATaDa is a powerful tool for understanding the regulation of chromatin during animal development. As untethered Dam is routinely used to normalise DamID data, this provides an opportunity for re-analysis of existing data, which may allow insights into cell-specific chromatin regulation. Furthermore, the fidelity to which CATaDa is able to identify accessible sequences underscores the necessity for the use of proper controls in conventional DamID experiments, so that local changes in open chromatin can be properly taken into account when considering transcription factor binding.

A recent study has further demonstrated the power of using Dam methylation for assaying chromatin accessibility in *Drosophila* (Sen et al., 2019). CATaDa was used to determine sites of differentially accessible chromatin in two separate neural stem cell populations. These data were compared with TaDa data for binding of the transcription factor, *Hunchback*, to shed light on the mechanisms by which neuronal diversity is generated. Together, these data indicate that chromatin state is imparted by early-acting spatial factors that establish accessible sites on which later temporal factors can act to specify the subsequently generated neuronal lineage. This study is also notable because the neural stem cell lineages that were profiled contained only 264 cells per embryo. The TaDa/CATaDa approach allowed for the simultaneous profiling of transcription factor binding and chromatin accessibility without having to dissociate and sort individual low-abundance cells from the whole embryo.

Chromatin accessibility is an important regulator of gene expression; however, another layer of complexity is added when you consider other elements, such as histone modifications. By examining the genome-wide occupancy of factors that are associated with histone modification signatures and nucleosome remodelling, chromatin can be broadly classified into five discrete states (red, yellow, green, blue and black) that correspond to active or repressive gene environments (Filion et al., 2010). Blue and green represent heterochromatin (largely silent), whereas yellow and red chromatin are associated with active gene expression. Black chromatin represents a third (and most prevalent) type of repressive environment, which lacks traditional heterochromatin markers. These chromatin states can be determined by the use of proxy proteins (Brahma, Pol II, HP1, Polycomb and Histone H1 for red, yellow, green, blue and black, respectively). By using TaDa to determine the binding of these proxy proteins, chromatin state maps of multiple cell types can be determined *in vivo* (Marshall and Brand, 2017). The chromatin landscape of neural stem cells and their progeny revealed that novel transitions between chromatin states underlie neural development. Together, CATaDa and chromatin profiling allow identification of regulatory elements and chromatin transitions in cell types in which chromatin dynamics were previously unknown.

## A new approach to identifying cell-type-specific RNA-chromatin interactions

Long non-coding RNAs (lncRNAs) are transcripts that are longer than 200 bp that can have roles in transcriptional regulation. Many interact with transcription factors and chromatin remodelling factors to influence gene expression at specific genomic loci (Marchese et al., 2017). lncRNAs are expressed in spatially and temporally restricted patterns *in vivo*. RNA pulldowns from cross-linked chromatin allow identification of the binding sites of lncRNAs in cell culture, but because of the requirement for tens of millions of cells their application *in vivo* has been limited (Chu et al., 2015). RNA-DamID is a novel approach for identifying the genomic occupancy of lncRNAs *in vivo* in a cell-type-specific fashion (Cheetham and Brand, 2018). RNA-DamID involves the genetic tagging of the lncRNA of interest with MS2, a stable hairpin that is derived from the MS2 bacteriophage. Dam is then fused to the MS2 Coat Protein (MCP), which binds to the MS2 RNA tag with nanomolar efficiency. The tagged lncRNA and Dam-MCP fusion can be expressed in a cell type of interest, in which Dam-MCP is recruited to sites of lncRNA-chromatin interaction and methylates nearby GATC motifs, thereby identifying cell-type-specific lncRNA-binding sites *in vivo* (Fig. 2D). RNA-DamID is far more sensitive than alternative pulldown-based approaches to map lncRNA-chromatin interactions such as ChART (Simon et al., 2011), RAP (Engreitz et al., 2013) and ChIRP (Chu et al., 2011), and has been used in as few as 30,000 neural stem cells. Importantly, RNA-DamID also appears to have fewer false positives than ChIRP. RNA-DamID can identify the binding sites of ectopically expressed (*roX2*) and endogenously tagged (*roX1*) lncRNAs, but has not yet been applied to other lncRNAs. Although RNA-DamID requires transgenesis, as do all DamID approaches, it is currently the only method that can resolve RNA-binding profiles from restricted cell populations *in vivo*. Thus, RNA-DamID will be an invaluable tool for identifying the mechanisms through which lncRNAs control gene expression and chromatin structure.

## Mapping the genomic co-occupancy of transcription factor complexes using split DamID

Transcription factors assemble into macromolecular complexes on chromatin. To understand the regulation of gene expression by transcription factors and their cofactors it is important to identify their occupied sites. Sequential ChIP-seq that targets multiple epitopes can be used to achieve this, but also relies upon specific antibodies and requires millions of cells. Alternatively, split DamID can profile the co-occupancy of two proteins at genomic loci (Hass et al., 2015). In this technique, the Dam methylase is split into two halves, with each half fused to one of two proteins of interest. When the two proteins are in close proximity, Dam is reconstituted and methylates nearby GATC sites (Fig. 2E). This approach has been used to identify Notch-binding sites that are co-occupied by the cofactors RBPJ, MAML (MAML1) and p300 (Ep300) in mouse kidney cells (Hass et al., 2015). Crucially, split DamID shares many of the advantages of DamID-related approaches that have been described previously, namely that it can be performed with little starting material (<10,000 cells) and does not require antibodies. Furthermore, split DamID could be spatially and temporally controlled, and could thus provide a useful tool for the identification of protein-protein interactions at specific genomic loci *in vivo*. However, so far, no subsequent studies have employed split DamID so it is unclear whether it can be generally applied to other co-occupying factors. Moreover, this technique currently lacks a control that takes into account the effect of open chromatin on transcription factor binding. It may be necessary to develop controls in which untethered Dam is reconstituted to give greater confidence in the co-bound loci that are identified.

## Identification of long-range chromatin interactions

The control of gene expression is dependent on long-range interactions of regulatory elements that are mediated by the formation of chromatin loops. Chromosome conformation capture (3C)-based experiments are currently the most widely used methods for studying chromosome conformation. Dam has also been used to determine long-range interactions (Cléard et al., 2014). Tethering Dam to a specific locus results in local methylation, as well as methylation of distant loci that are in close three-dimensional proximity, thereby identifying long-range DNA interactions (Fig. 2F). First used in yeast cells, this approach can detect interactions between telomeres and silencer sequences (Lebrun et al., 2003). In this case, Dam was targeted to Tet operator sequences by fusing with TetR. More recently, a similar approach has been employed in mouse embryonic stem cells to observe and validate long-range interactions (Redolfi et al., 2018 preprint). Using high-throughput sequencing to detect methylated DNA, this study yielded results that are comparable with 4C (a 3C-derived technique in which multiple interactions with a single locus can be detected). Furthermore, adding Tet operators at multiple widely spaced genomic loci, allows for broader interrogation of chromosome conformation in a single experiment. A similar approach makes use of the GAL4/UAS system, in which Dam-GAL4 is targeted to UAS in *Drosophila* (Cléard et al., 2006, 2014). This approach has been used to identify chromatin loops formed in the homeotic bithorax complex.

Although these methods have proven effective for chromosome conformation studies, they require the introduction of recognition sites at defined points in the genome. Therefore, they may not be well suited for *in vivo* studies on organisms that are more difficult to manipulate genetically. Synthetic transcription factors, such as engineered zinc fingers or transcription activator-like effectors (TALEs), can be made to recognise any DNA sequence (Bogdanove and Voytas, 2011). Dam has been fused to custom TALE proteins to detect changes in long-range interactions in the mouse prefrontal cortex (Mitchell et al., 2016). This approach has the advantage of being able to supply all the necessary components by injection, so may be better suited to mammalian studies.

The use of transgenic Dam fusions allow for the use of smaller amounts of tissue and analysis of chromosome architecture *in vivo*, in a cell type of interest. Importantly, these approaches do not rely on crosslinking or ligation (as with 3C-based methods), so can be used for independent *in vivo* validation of chromatin loops. Therefore, these methods have the potential to be valuable in understanding how gene regulation is affected by 3D chromatin structure during both animal development and physiological changes.

## DamID in single cells

### Chromatin interactions in single cells

Understanding the nuclear dynamics of individual cells has been an ambition in molecular biology for decades. In 2015, the first genome-wide maps of a chromatin protein binding in a single cell were reported using a modified DamID protocol (Kind et al., 2015). This study determined the binding sites of the nuclear lamina-associated protein lamin B1 in single human cells, which highlighted the variability of lamin-associated domains between cells. One limitation of this approach is that it is not possible to use a conventional Dam-only control to normalise binding data. Therefore, it may be difficult to distinguish between open chromatin and bona fide transcription factor-binding sites. In addition, lamin B1 interacts with very large domains, for which high resolution is not required. For a factor that binds much smaller genomic regions such as a transcription factor, single cell DamID may be difficult.

Determining transcription factor binding in single cells is currently extremely challenging. To date, only single cell DamID and a ChIP-based approach (Rotem et al., 2015) have accomplished this, and neither has been validated by subsequent studies. Despite the fact that single cell DamID is in its infancy, strong interest in single cell studies (Kelsey et al., 2017) is likely to drive further development of the technique, which has the potential to yield important insights into transcription factor-binding heterogeneity between individual cells.

### Single cell live imaging of chromatin interactions

Visualisation of the dynamics of chromatin protein binding at single cell resolution is also possible with DamID (Kind et al., 2013). Using GFP that has been fused to a catalytically inactive restriction endonuclease DpnI fragment (that binds methylated GATCs) enables the visualisation of binding sites within the nucleus immediately after methylation (Fig. 2G). Furthermore, the stability of the m6A modification can be exploited to track the intracellular movement of methylated loci live using the GFP-DpnI fusion. This technique is referred to as m6A-tracer. So far, this approach has been used to understand chromatin interactions with the nuclear lamina, but it could be extended to visualise the dynamics of methylated DNA that has been generated using any Dam-fusion protein.

## Techniques that improve upon methylation resolution

One limitation of DamID is the low resolution, which is restricted by the availability of genomic GATC motifs. Dam immunoprecipitation (DamIP) makes use of a point mutation in Dam (DamK9A) that reduces the recognition site to ATC (Xiao et al., 2010). As ATC motifs occur much more frequently in the genome, expression of DamK9A can yield much higher-resolution data. Alternatively, methyl adenine identification (MadID) employs an alternative adenine methylase, EcoGII, which has been used to determine the binding sites of lamin B1 (Sobecki et al., 2018). Unlike Dam, EcoGII has no motif constraints, which allows unprecedented resolution and information from regions of the genome that are devoid of GATC sequences. Importantly, because there are no restriction endonucleases that specifically recognise adenine methylation outside of a GATC context, both DamIP and MadID require the pull down of methylated DNA with an m6A-specific antibody. However, caution must be advised; m6A antibody pulldowns require appropriate controls to avoid off-targets (Lentini et al., 2018), and the potential for greater toxicity that results from more extensive methylation has not been fully investigated (see Box 2). These approaches could be used to increase the resolution of the diverse range of DamID applications that are discussed in this review.

## Future perspectives

Despite remarkable progress in understanding the mechanisms of gene regulation in cell culture, insights into chromatin interactions in native tissues have lagged behind. The recent developments in DamID-based chromatin profiling techniques now enable us to address biological questions that have been previously out of reach. Assays for transcription factor binding, RNA-chromatin interactions, or chromatin accessibility have frequently used heterogeneous tissues, and although these experiments have been informative, they do not capture the diversity of gene regulation across cell types. By using cell-type-specific DamID methods we can now easily study how epigenomic features regulate transcriptional diversity – spatially and temporally – in small populations of cells within heterogeneous tissues (as summarised in Table 1).

**Table 1.**
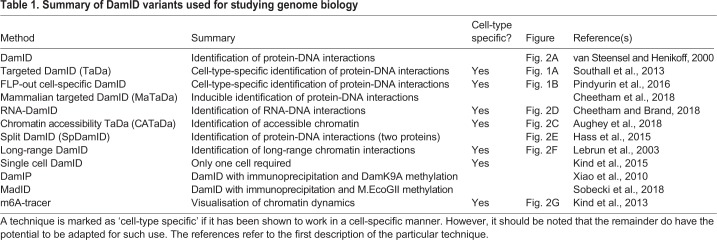
**Summary of DamID variants used for studying genome biology**

As most of these methods require much smaller amounts of tissue than alternative methods, Dam-related approaches may be useful for assaying chromatin interactions when samples are difficult to obtain in large quantities, such as a small population of cells within a larger tissue or whole embryo. For example, one recent study identified Pol II-bound genes in the Kenyon cells of the *Drosophila* mushroom body. There are ∼2500 of these cells in a brain of over 100,000 cells, so this represents ∼2.5% of the cell population (Widmer et al., 2018). Moving forward, DamID will prove invaluable for profiling restricted cell populations in animals (Aughey and Southall, 2016). DamID may also be extended to organoid cultures, which are emerging as important models for understanding development and disease.

An advantage of DamID technologies is that, regardless of the method that is used to generate the methylated DNA, the subsequent detection of these sequences uses a standard set of reagents (see Box 1). Therefore, each experiment can be performed with minimal optimisation and without requiring high-quality antibodies. Thus, performing screens to assay multiple chromatin-associated factors, cells or time points is an ideal application for DamID. Indeed, this has already been achieved in cell culture, in which the binding profiles of dozens of chromatin proteins have been generated using DamID in a single study (van Bemmel et al., 2013).

Several DamID-based methods could be combined to allow an even broader range of biological problems to be approached. For example, an MS2-tagged lncRNA could be co-expressed with a transcription factor using the split DamID technique to assay co-occupancy of a transcript and a protein in a single cell, or imaging of lncRNA-chromatin associations could be achieved using RNA-DamID and the DpnI-GFP fusion. As Dam can be tethered to any protein of interest, it is likely that the range of DamID approaches will expand as new molecular biology tools become available. Dam could be combined with novel synthetic binding proteins or transcription factors, which would provide researchers with even greater flexibility to address biological questions *in vivo*. Therefore, DamID offers an incredibly flexible portfolio of tools for studying chromatin biology. Furthermore, Dam may be employed to generate novel biological properties in synthetic biology. This idea is exemplified by a recent study, which showed that Dam is recruited to specific loci to create a synthetic epigenetic mark that is recognisable by the DpnI-binding domain fused to a transcriptional effector (Park et al., 2019). This results in reliable transactivation or repression at target loci with epigenetic properties such as heritability and spatial propagation.

Through the creativity of individual researchers, the potential of DamID will continue to grow and novel applications for DamID technologies, beyond those discussed here, will likely be developed. With advances in DamID techniques, combined with the ability to manipulate genetic regulatory elements with CRISPR, the functional dissection of chromatin interactions and regulatory elements is now possible, which will enable a greater understanding of gene regulation *in vivo*.
